# Rhino-orbito-cerebral mucormycosis (ROCM) with internal carotid artery stenosis in a diabetic patient with caries tooth and oroantral fistula

**DOI:** 10.1259/bjrcr.20150447

**Published:** 2016-05-02

**Authors:** Kataveeranahally Shekar Manjunath, Santosh Shivaswamy, Jayashree Dattatraya Kulkarni, Raghavendra Kenkare Venkatachalaiah

**Affiliations:** ^1^ Department of Radiology, Columbia Asia Hospital, Bangalore, India; ^2^ Department of Otorhinolaryngology, Columbia Asia Hospital, Bangalore, India; ^3^ Department of Pathology, Columbia Asia Hospital, Bangalore, India

## Abstract

Mucormycosis is a rare, potentially fatal and opportunistic infection caused by fungi belonging to the order Mucorales. Rhinocerebral, gastrointestinal, pulmonary, cutaneous and disseminated are the different forms of mucormycosis. Rhinocerebral mucormycosis is the most common type and presents as a highly destructive infection in immunocompromised hosts, especially in patients with poorly controlled diabetes. The infection originates in the nasal mucosa owing to fungal inoculation and then spreads to the paranasal sinuses, orbits, orbital apex, cavernous sinuses and brain. Our patient was a 36-year-old female with poorly controlled diabetes who presented with orbital symptoms and signs, with very subtle involvement of the sinuses. She had stenosis of the entire left internal carotid artery, with multiple small infarcts in the left frontal and parietal lobes. She incidentally had tooth caries tooth with a periapical cyst and an oroantral fistula. Ours was a histopathologically proven case of rhino-orbito-cerebral mucormycosis.

## Clinical history and laboratory findings

**Figure 1.**
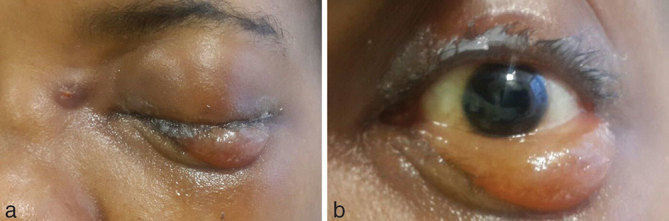
The patient's orbital area photographs show left periorbital oedema and swelling on the dorsal aspect of the root of the nose (a), lower conjunctival oedema with mild proptosis of the mucosa and a fixed and dilated pupil (b).

A 36-year-old female presented with gradually increasing left eye swelling, progressive loss of vision and left-sided facial pain for 2 months. A few days prior to the peaking of symptoms, she had symptoms of cold. She was a known diabetic and hypertensive. On examination (Figure 1a,b), there was left periorbital oedema, swelling on the dorsal aspect of the root of the nose and lower conjunctival oedema with mild proptosis of the mucosa. The patient was unable to open the left eyelid completely. She could not perform any ocular movements. The pupil was fixed and dilated. On ophthalmoscopy, the disc appeared pale. Blood sugar level at the time of admission was 280 mg dl^–1^. The surgeon suspected left paranasal sinus pathology with orbital extension and requested a CT scan of the paranasal sinuses and an MRI of the orbits with screening of the brain.

## Imaging findings

**Figure 2.**
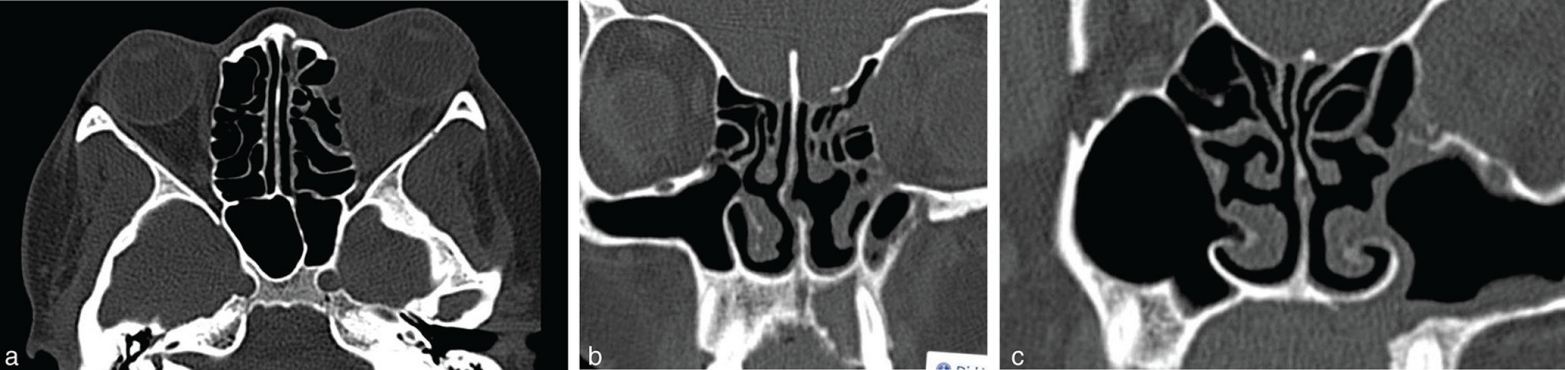
(a–c) Axial and coronal CT images of the paranasal sinuses and orbits demonstrate mild mucosal thickening of the left maxillary (c) and left ethmoid (a, b) sinuses, proptosis of the left globe, retro-orbital fat stranding, soft tissue thickening along the medial wall of the orbit and subtle bony erosion in the left lamina papyracea (a), bony defect in the alveolar margin of the maxilla at the root of the left incisor tooth (b) and an oroantral fistula (c).

CT scan of the paranasal sinuses (Figure 2a–c) demonstrated mild mucosal thickening of the left maxillary and ethmoid sinuses, bony defect in the alveolar margin of the maxilla at the root of the left incisor tooth and an oroantral fistula. CT scan of the orbital region demonstrated proptosis of the left globe, retro-orbital fat stranding and soft tissue thickening along the medial wall of the orbit with subtle bony erosion in the left lamina papyracea.

### MRI of the orbits

**Figure 3.**
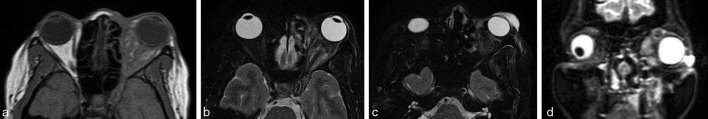
Axial *T*_1 _weighted images show fat stranding in the retro-orbital, orbital apex and left cavernous sinus regions with minimal proptosis of the left globe (a). Axial *T*_2_ weighted fat-saturated images (b, c) show extensive fat stranding in the retro-orbital region, ill-defined isointense to hypointense soft tissue along the medial wall of the left orbit and intraconal retro-orbital space extending to the orbital apex. On short tau inversion-recovery coronal images (d), the extraocular muscles appear oedematous. Left internal carotid artery flow void is small in calibre with adjacent soft tissue thickening in the left cavernous sinus (a–c). A small focal hyperintense collection is seen in the left preseptal space (c, d).

*T*_1_ weighted images (Figure 3a) demonstrated fat stranding in the retro-orbital, orbital apex and left cavernous sinus regions, with minimal proptosis of the left globe.

*T*_2_ weighted fat-saturated axial images (Figure 3a,b) and short tau inversion-recovery coronal images (Figure 3d) demonstrated extensive fat stranding in the retro-orbital region; ill-defined isointense to hypointense soft tissue along the medial wall of the left orbit and the intraconal retro-orbital space, which extended to the orbital apex. The extraocular muscles appeared oedematous. Left internal carotid artery flow void was small in calibre with adjacent soft tissue thickening in the left cavernous sinus. A small focal hyperintense collection was seen in the left preseptal space.

**Figure 4.**
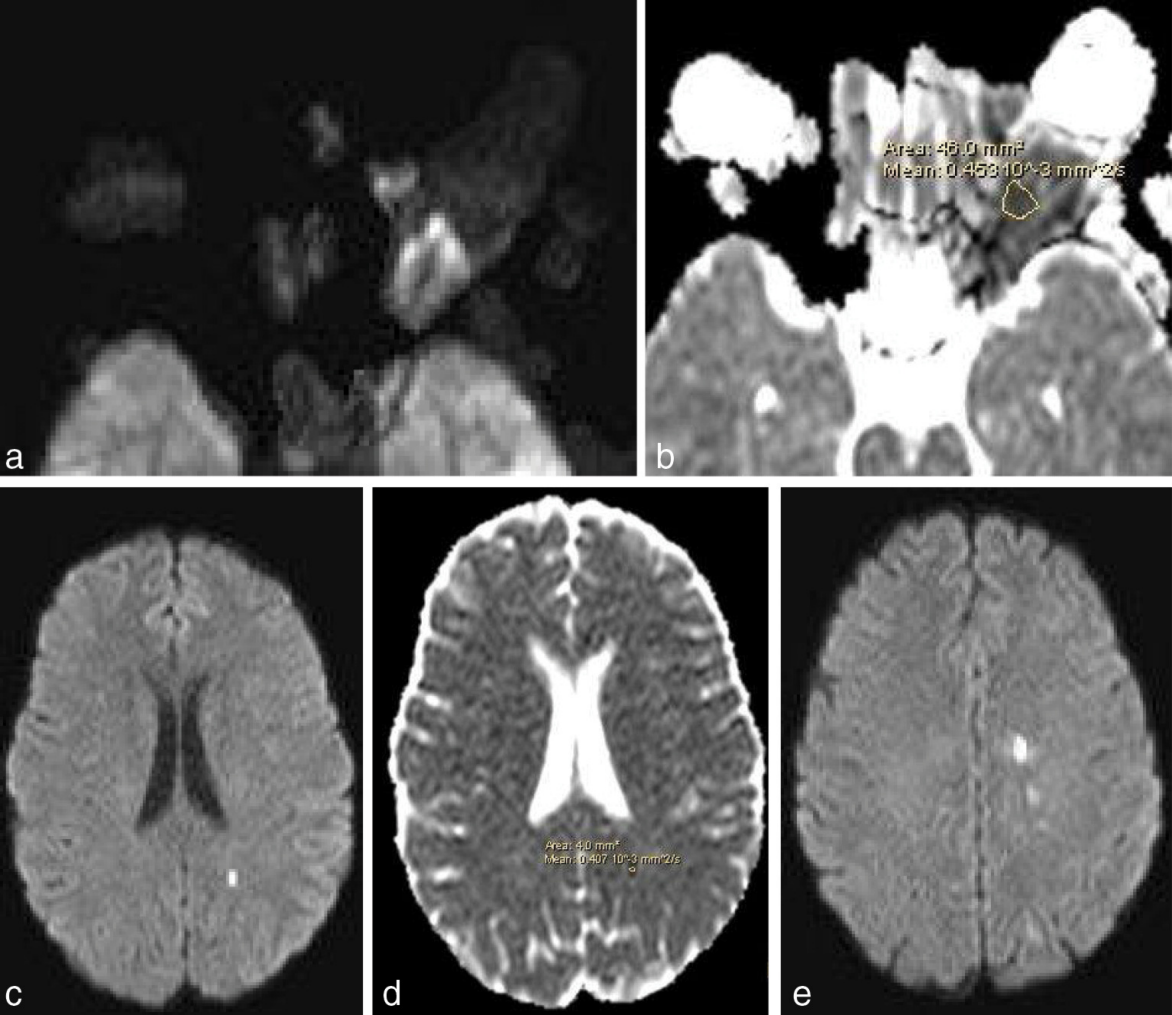
Diffusion-weighted images show restricted diffusion in the two intraorbital lesions (including the encased optic nerve) with very low apparent diffusion coefficient values (0.45 × 10^–3^ mm^2^ s^–1^). The preseptal lesion does not show any restriction of diffusion (a, b). Multiple small acute infarcts are seen in the left corona radiata and left parietal lobe with apparent diffusion coefficient value similar to the orbital lesions (0.407 × 10^–3^ mm^2^ s^–1^) (c–e).

Diffusion-weighted images (Figure 4a–e) showed restricted diffusion in the two intraorbital lesions (including the encased optic nerve) with low apparent diffusion coefficient values (0.45 × 10^–3^ mm^2^ s^–1^). The preseptal lesion did not show any restriction of diffusion. Multiple small acute infarcts were seen in the left corona radiata and parietal lobe with an apparent diffusion coefficient value similar to the orbital lesions (0.407 × 10^–3^ mm^2^ s^–1^).

**Figure 5.**
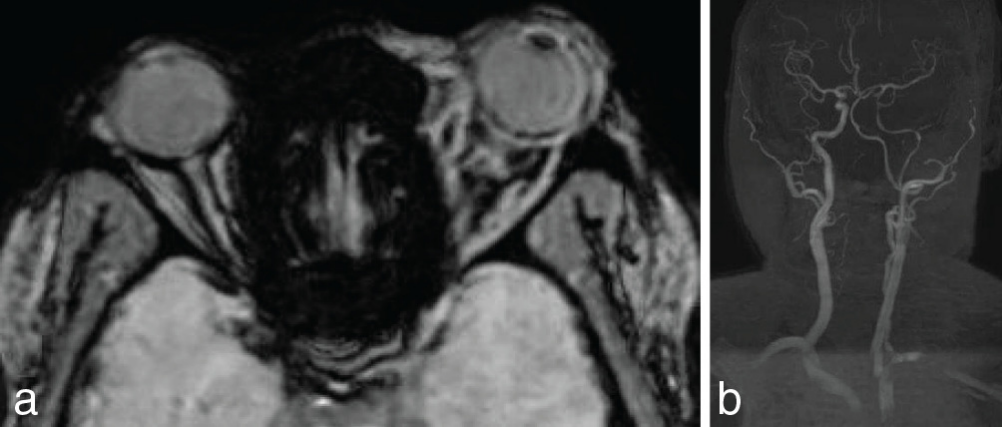
Susceptibility weighted imaging demonstrates blooming in the two intraorbital images (a). MR angiography demonstrates narrowing of the left internal carotid artery from its origin, involving both the intracranial and the extracranial portions (b).

Susceptibility weighted imaging (Figure 5a) demonstrated blooming in the two intraorbital images. MR angiography (Figure 5b) demonstrated narrowing of the left internal carotid artery from its origin, involving both the intracranial and extracranial portions.

**Figure 6.**
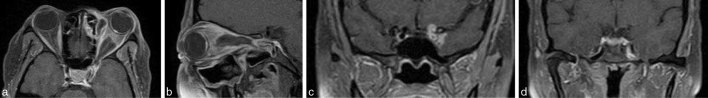
Post-contrast *T*_1_ weighted fat-saturated axial, sagittal and coronal images demonstrate three peripherally enhancing lesions in the left orbit. A 15 × 8 × 10 mm lesion is seen in the extraconal space on the medial aspect of the orbit subperiosteally, displacing the medial rectus laterally with subtle enhancement in the left ethmoid sinus (a). A 33 × 18 × 15 mm intraconal retro-orbital lesion communicates with the lesion along the medial wall and extends to the orbital apex and left cavernous sinus, with the intracranial component measuring 7 mm (a, c). The left optic nerve is encased by the intraconal lesion with enhancement in the distal part of the optic nerve close to insertion at the globe (a, b). The enhancement is seen to extend along the left internal carotid artery, which has a small calibre with thickened wall (a–d). There is also an inferior extension into the left pterygopalatine fossa. The third lesion is seen in the lower preseptal space measuring 23 × 7× 9 mm, and shows signal intensity similar to fluid with peripheral enhancement (b).

Post-contrast fat-saturated *T*_1_ weighted images (Figure 6a–d) demonstrated three peripherally enhancing lesions in the left orbit. A 15 × 8 × 10 mm lesion was seen in the extraconal space on the medial aspect of the orbit subperiosteally, displacing the medial rectus laterally with subtle enhancement in the left ethmoid sinus (Figure 6a). A 33 × 18 × 15 mm intraconal retro-orbital lesion was seen communicating with the lesion along the medial wall and extending to the orbital apex and the left cavernous sinus, with the intracranial component measuring 7 mm (Figure 6a,c). The left optic nerve was encased by the intraconal lesion with enhancement in the distal part of the optic nerve close to its insertion into the globe (Figure 6a,b). The enhancement was seen to extend along the left internal carotid artery, which had a small calibre with a thickened wall (Figure 6a–d). There was also inferior extension into the left pterygopalatine fossa (Figure 6b). The third lesion was seen in the lower preseptal space measuring 23 × 7 × 9 mm and showed signal intensity similar to fluid with peripheral enhancement.

## Perioperative and histopathological findings

Surgical endoscopic excision of the orbital lesion was performed. Intraoperatively, cheesy white material with whitish spots was noted. It was thick in consistency and showed minimal bleeding on dissection.

**Figure 7.**
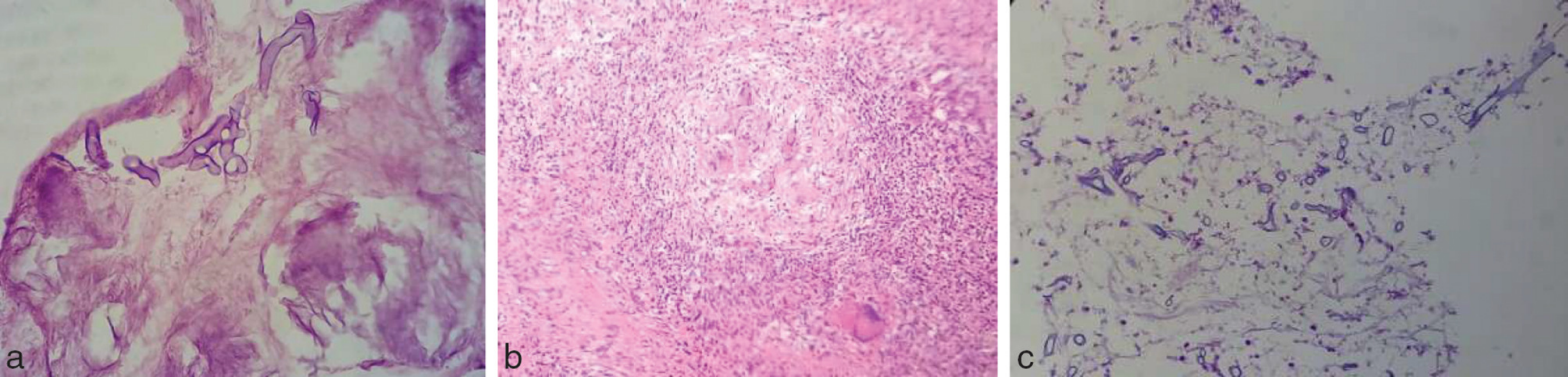
Haematoxylin and eosin staining of tissue samples from the left orbit and orbital apex show broad, aseptate, obtuse-angle branching hyphal elements amid necrotic tissue when viewed with 40× magnification (a). They also show epithelioid granuloma with giant cells and lymphocyte predominant inflammatory cells when viewed with 10× magnification (b). The presence of fungal elements is confirmed with periodic acid–Schiff staining when viewed with 10× magnification (c).

Tissue samples from the left orbit and orbital apex on haematoxylin and eosin staining showed necrotic tissue with inflammatory cells composed of neutrophils, lymphocytes, plasma cells, histiocytes and a few giant cells amid which were seen broad, aseptate, obtuse-angle branching hyphal elements (Figure 7a,b). The presence of fungal elements was confirmed with periodic acid–Schiff staining (Figure 7c).

Tissue samples from the left ethmoid and maxillary sinuses demonstrated moderate chronic inflammatory cell infiltrate and fragments of bony lamellae. Tissue samples from the left ethmoid sinus demonstrated epithelioid granuloma with giant cells. However, acid-fast bacillus stain and culture were negative and so tuberculosis was ruled out.

## Discussion

Paltauf^1^ was the first to describe mucormycosis in 1885. It is caused by the fungal organisms of the family of Mucoraceae (*Mucor, Rhizopus* and *Absida*) belonging to the class of Phycomycetes. They are recognized microscopically as broad, non-septate and obtuse-angle branching hyphae. They are ubiquitous and normally saprophytic in humans. Inoculation occurs when spores reach the nasal cavity during inhalation. They can produce fatal disease in persons with immunosuppression.

Five clinical forms of mucormycosis are recognized: rhino-orbito-cerebral, gastrointestinal, pulmonary, cutaneous and disseminated. The rhinocerebral form is the most common and is seen mostly in patients with uncontrolled diabetes.^2^

In the rhinocerebral form, the infection originates in the nasal cavity and then spreads to the paranasal sinuses. From the sinuses, it spreads to the medial orbit and the orbital apex through the nasolacrimal duct and the medial orbital wall. Spread of infection from the sinuses to the orbit is facilitated by the thinness of the lamina papyracea, congenital dehiscence in the medial wall and fenestrations in the medial wall by arteries and veins.^3^ It spreads to the brain *via* the orbital apex, orbital vessels or the cribriform plate.^4^ In our case, there was involvement of the left orbit along the medial wall, the preseptal space and the orbital apex, with subtle involvement of the left ethmoid sinus.

Intracranial spread to the cavernous sinus may result in haemorrhage or thrombosis of the internal carotid artery, or thrombosis of the cavernous sinus.^5^ In our case, there was involvement of the cavernous sinus, stenosis of the left internal carotid artery and a small peripherally enhancing lesion just above the cavernous portion of the internal carotid artery. Internal carotid artery occlusion has a high mortality rate.^6^ From the cavernous sinus, the infection can spread to the brain parenchyma or the cranial nerves such as the fifth and the seventh. Inferiorly, the infection can spread to the skull base, including the pterygopalatine fossa, as in our case. Brain parenchyma involvement may be in the form of infarcts owing to vascular thrombosis, mycotic emboli and frontal lobe abscesses.

The pathophysiology of mucormycosis is related to the invasion of the walls of the vessels, especially the arteries. Hyphae could be demonstrated in the walls of the internal carotid arteries in all the cases in a study by Lowe and Hudson.^6^ Thrombosis may be due to endothelial damage from hyphal invasion or growth within the lumen.

The common presenting symptoms are headache, low-grade fever, facial swelling, sinusitis, orbital apex syndrome and cranial palsies from cavernous sinus involvement. Visual loss occurs much earier in rhinocerebral mucormycosis than in bacterial cavernous sinus thrombosis.^7^ Differential diagnosis for orbital cellulitis and sinusitis in patients with immunosuppression are: mucormycosis and other fungal infections, paranasal sinus malignancy, bacterial cellulitis, carotid cavernous fistula and thrombosis, inflammatory pseudotumor and Graves’ disease.^8^ Rhinocerebral mucormycosis should be considered in the differential diagnosis of patients presenting with symptoms of unilateral cranial nerve involvement, as in Garcin syndrome.^9^

CT scan findings described in sinonasal mucormycosis include soft tissue opacification of the sinuses with hyperdense material, nodular mucosal thickening and absence of fluid levels.^10,11^ Maxillary, ethmoid, frontal and sphenoid sinuses are involved in decreasing order of frequency.^10^ Our case demonstrated mucosal thickening in the maxillary sinus without any nodularity, hyperdensity or air–fluid levels. Orbital images in our case demonstrated thickening and lateral displacement of the medial rectus muscle, which is characteristic of orbital invasion from disease in the ethmoid sinuses.^11^ Lack of enhancement of the ophthalmic and internal carotid arteries or the superior ophthalmic vein can occur owing to vasculitis and thrombosis.^12^

MRI has a very important role in the diagnosis of rhinocerebral mucormycosis, especially in the early detection of orbital and intracranial complications.

Sinus contents can show variable MR signal characteristics on *T*_1_ and *T*_2_ weighted images, with majority of the lesions showing isointense signal relative to brain in *T*_1_ weighted images.^13^ The signal intensity in *T*_2_ weighted images has been reported to be more variable, with 20% showing hyperintense signal and the rest of the lesions showing either hypointense or isointense signal.^13^

Diffusion-weighted image sequences may demonstrate restriction of diffusion in the lesions owing to infarction.^9^ Diffusion restriction may be the earliest indicator of optic nerve infarction on MRI.^14^

Contrast-enhanced *T*_1_ weighted images better demonstrate the intracranial spread by recognizing meningeal enhancement, invasion of the cavernous sinus or the internal carotid artery, and vascular complications such as ischaemia.^14,15^ The enhancement pattern may be variable.^8^ Rhinocerebral mucormycosis should be suspected when there is a lack of enhancement of the mucosa of the sinus owing to invasion of smaller vessels supplying the mucosa by the hyphae.^9^ Non-enhancement of the involved turbinate is called the “black turbinate sign”.^9^

In our case, there were multiple peripherally enhancing lesions in the orbit with a small intracranial extension. Diffuse enhancement was seen in the retro-orbital space, optic nerve, along the intracranial portion of the internal carotid artery and the cavernous sinus. Subtle enhancement was seen in the left ethmoid sinus in continuity with enhancement along the medial orbital wall. The entire internal carotid artery showed moderate stenosis.

Prognosis is very poor and mortality is very high in rhinocerebral mucormycosis with intracranial extension.

## Conclusions

We want to emphasize that MRI is the most important imaging modality in making an early diagnosis and detecting complications. CT scan is complimentary to MRI and is helpful in detecting any bony defects. Histopathological identification of broad, aseptate, obtuse-angle branching hyphal elements in the background of inflammatory necrotic tissue is confirmatory. Our case is unique as there was extensive orbital involvement, stenosis of the entire internal carotid artery and very subtle involvement of the ethmoid sinus. Although biopsy from the ethmoid sinus did not reveal any fungal elements, we presume that it was owing to a lack of representative sample. Our case is also unique because of the presence of tooth caries, with periapical cyst and oroantral fistula. We are not sure whether these are incidental findings or related to the spread of mucormycosis.

## Learning points

1. Mucormycosis is caused by fungal organisms belonging to the family of Mucoraceae (*Mucor, Rhizopus* and *Absida*) that are ubiquitous and normally saprophytic in humans but can produce fatal disease in persons with immunosuppression. The causative fungi are recognized by viewing broad, aseptate, obtuse-angle branching hyphal elements.
2. Five major types of mucormycosis are recognized: rhinocerebral, gastrointestinal, pulmonary, cutaneous and disseminated. The rhinocerebral form is the most common and is seen mostly in patients with uncontrolled diabetes.
3. In the rhino-orbito-cerebral form, the infection originates in the nasal cavity and spreads to the paranasal sinuses, orbits and then intracranially, with cerebral or cranial nerve involvement. Intracranial spread to the cavernous sinus may result in haemorrhage or thrombosis of the internal carotid artery, or thrombosis of the cavernous sinus.
4. The pathophysiology of mucormycosis is related to the invasion of the walls of the vessels, especially the arteries.
5. The common presenting symptoms are headache, low-grade fever, facial swelling, sinusitis, orbital apex syndrome, visual loss, cranial palsies from cavernous sinus involvement and focal neurological defects owing to cerebral involvement.
6. The differential diagnoses for orbital cellulitis and sinusitis in patients with immunosuppression are: mucormycosis and other fungal infections, paranasal sinus malignancy, bacterial cellulitis, carotid cavernous fistula and thrombosis, inflammatory pseudotumour and Graves’ disease.
7. CT scan findings in sinonasal mucormycosis are soft tissue opacification of the sinuses with hyperdense material, nodular mucosal thickening and an absence of fluid levels.
8. The maxillary, ethmoid, frontal and sphenoid sinuses are involved in decreasing order of frequency.
9. MRI has a very important role in the diagnosis of rhinocerebral mucormycosis, especially in the early detection of orbital and intracranial spread and their complications. Lesions can show variable MR signal characteristics on *T*_1_ and *T*_2_ weighted images, variable contrast enhancement and restriction of diffusion. Contrast-enhanced *T*_1_ weighted images are helpful in delineating the orbital and intracranial spread.
10. Prognosis is very poor and mortality is very high in rhino-orbito-cerebral mucormycosis, especially with intracranial extension.

## Consent

Informed consent was obtained and is held on record.
